# Oligoorganogermanes: Interplay between Aryl and Trimethylsilyl Substituents

**DOI:** 10.3390/molecules27072147

**Published:** 2022-03-26

**Authors:** Kirill V. Zaitsev, Oleg Kh. Poleshchuk, Andrei V. Churakov

**Affiliations:** 1 Department of Chemistry, M.V. Lomonosov Moscow State University, Leninskie Gory 1, 3, 119991 Moscow, Russia; 2 A.V. Topchiev Institute of Petrochemical Synthesis, Russian Academy of Sciences, Leninskii Prospekt 29, 119991 Moscow, Russia; 3 Faculty of Chemistry, National Research Tomsk State University, Lenin Avenue 36, 634050 Tomsk, Russia; poleshch@tspu.edu.ru; 4 Department of Chemistry, Tomsk State Pedagogical University, Kievskaya Street 60, 634061 Tomsk, Russia; 5 N.S. Kurnakov Institute of General and Inorganic Chemistry, Russian Academy of Sciences, Leninskii Prospekt 31, 117901 Moscow, Russia; churakov@igic.ras.ru

**Keywords:** organogermanium compounds, oligoorganogermanes, donor–acceptor molecules, element–element bond, group 14 elements, σ-conjugation, single-crystal XRD analysis, UV/vis absorption, DFT calculations, main group metal chemistry

## Abstract

Derivatives of main group elements containing element–element bonds are characterized by unique properties due to σ-conjugation, which is an attractive subject for investigation. A novel series of digermanes, Ar_3_Ge-Ge(SiMe_3_)_3_, containing aryl (Ar = *p*-C_6_H_4_Me (**1**), *p*-C_6_H_4_F (**2**), C_6_F_5_ (**3**)) and trimethylsilyl substituents, was synthesized by the reaction of germyl potassium salt, [(Me_3_Si)_3_GeK*THF], with triarylchlorogermanes, Ar_3_GeCl. The optical and electronic properties of such substituted oligoorganogermanes were investigated spectroscopically by UV/vis absorption spectroscopy and theoretically by DFT calculations. The molecular structures of compounds **1** and **2** were studied by XRD analysis. Conjugation between all structural fragments (Ge-Ge, Ge-Si, Ge-Ar, where Ar is an electron-donating or withdrawing group) was found to affect the properties.

## 1. Introduction

**Scheme 1:**
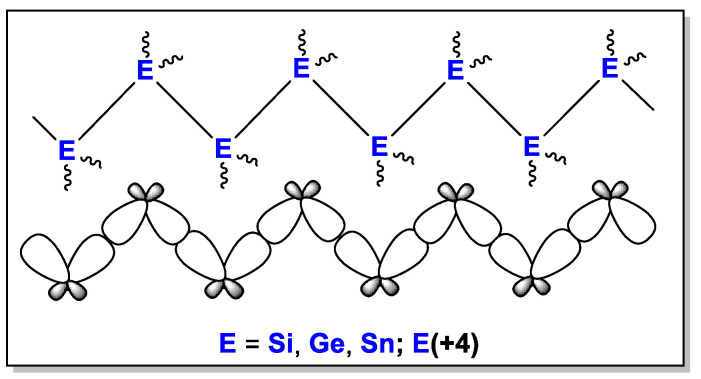
Schematic representation of oligoorganotetrelanes.

Research into organic derivatives of main group elements is a topical issue of organometallic chemistry [1,2,3,4]. Many efforts have been made to develop improved synthetic methods, determine new properties, find relationships between structures and properties, and produce novel materials. Some of the main group element derivatives are molecular compounds of group 14 elements (E = Si [5], Ge [6], Sn [7], Pb [8]) [9] containing element–element bonds (oligoorganotetrelanes). Unusual properties (UV/vis absorbance, luminescence, electrochemical activity, thermochromism, etc.) that appear in these compounds due to σ-conjugation [10] (Scheme 1) are of evident research interest [11].

Compounds of this type can be molecular or polymeric. Molecular derivatives can be regarded as models for polymers and “hybrid” materials based on them [12,13,14], including molecular conductors [15] and semiconductors [16]. Furthermore, catenated group 14 element derivatives can be used as synthetic reagents. Thus, Me_2_PhSi-GeEt_3_ have been applied recently in C-H activation [17] of arenes for synthesis of Ar-GeEt_3_ while (Me_3_Si)_3_SiR were used as radical precursors under photoredox conditions [18].

From the results of Weinert et al. [19,20] and Zaitsev et al. [21], aryl substituents are known to take part in σ-conjugation between Ge atoms in oligoorganogermanes (increasing by σ,π-conjugation the energy level of the highest occupied molecular orbital (HOMO)). Furthermore, the electron-donating or acceptor properties of the substituents also significantly determine the properties of catenated group 14 derivatives. At the same time, Marschner et al. have established for a wide range of molecular cyclic and linear trimethylsilyl and trimethylgermyl oligotetrelanes that the presence of sterically bulk EMe_3_ groups (E = Si, Ge; (Me_3_E)_3_Ge[EMe_2_]_n_Ge(EMe_3_)_3_, *n* = 0–3) [22,23,24] significantly affects the conformation-promoting *transoid* disposition of the substituents in the E-E chain [25], which results in a more significant σ-conjugation.

The aim of the present research work was to study in detail the catenated Ge compounds containing aryl and trimethylsilyl groups. Compounds of such type have been studied previously only slightly by Malella and Geanangel [26] and Zaitsev et al. [27]. Here, we present their directed synthesis, study their structures, investigate their optical properties by absorption spectroscopy and establish the main structure–property features by quantum chemistry (DFT) calculations. As typical examples, we choose Ar_3_Ge-Ge(SiMe_3_)_3_ (Ar = *p*-C_6_H_4_Me (**1**), *p*-C_6_H_4_F (**2**), C_6_F_5_ (**3**)), where the electronic properties of aryl groups change from donating to accepting (**1** and **2** vs. **3**). Interestingly, based on the electron-donating SiMe_3_ (electronegativity for Ge, 1.99–2.02; Si, 1.74–1.91) [28] and electron-withdrawing *p*-C_6_H_4_F, C_6_F_5_ properties of the substituents in **2** and **3**, both these compounds can be called donor–acceptor compounds [21,29].

## 2. Results and Discussion

### 2.1. Synthesis

**Scheme 2:**
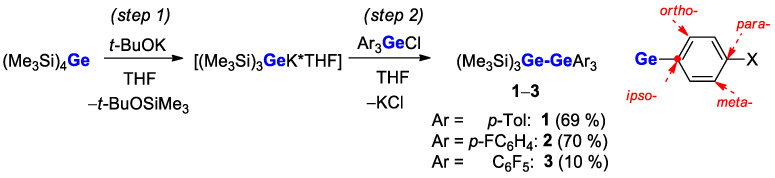
Synthesis of the digermanes studied.

The target digermanes were synthesized in two-step synthesis (Scheme 2). The first stage (step 1) includes the in situ preparation of THF solvated germyl-potassium salt, [(Me_3_Si)_3_GeK*THF], by treatment of (Me_3_Si)_4_Ge with *t*-BuOK in THF, using the procedure developed by Marshner et al. [30]. At the second stage (step 2), the corresponding electrophiles Ar_3_GeCl reacted with generated Ge nucleophile to give the products. The high yields (69–70%) were observed for compounds **1** and **2** (Ar = *p*-C_6_H_4_Me, *p*-C_6_H_4_F); in the case of **3** (Ar = C_6_F_5_), the yield decreased down to 10% due to side reactions, such as the substitution of F or C_6_F_5_ groups at the Ge atom by the strong germyl nucleophile [21]. Indeed, ^19^F NMR spectral analysis of the reaction mixture for **3** indicated the complex mixture of compounds, whereas pure product may be isolated only after chromatography. Interestingly, the developed chemistry for further substitution of SiMe_3_ groups in **1**–**3** opens the new perspective [31] for applying Ar_3_Ge-Ge(SiMe_3_)_3_ in the synthesis of functionalized compounds.

Compounds **1**–**3** were isolated as white powders with high solubility in all typical organic solvents including *n*-hexane, indicating their weak polarity and emphasizing yet again their analogy to alkanes. The compounds were characterized by NMR (^1^H, ^13^C, ^19^F) (Supplementary Materials, Figures S1–S11) and UV/visible (Supplementary Materials, Figures S12–S14) spectroscopy, elemental analysis and X-ray diffraction analysis.

### 2.2. NMR Spectroscopy

The main NMR spectral parameters for compounds **1**–**3** (CDCl_3_, RT) are quite similar; the presence of one set of signals for each compound is characteristic of molecules with a highly symmetric structure (*C_3v_* symmetry) in solution with free rotation of different molecular fragments.

### 2.3. XRD Structures

**Figure 1:**
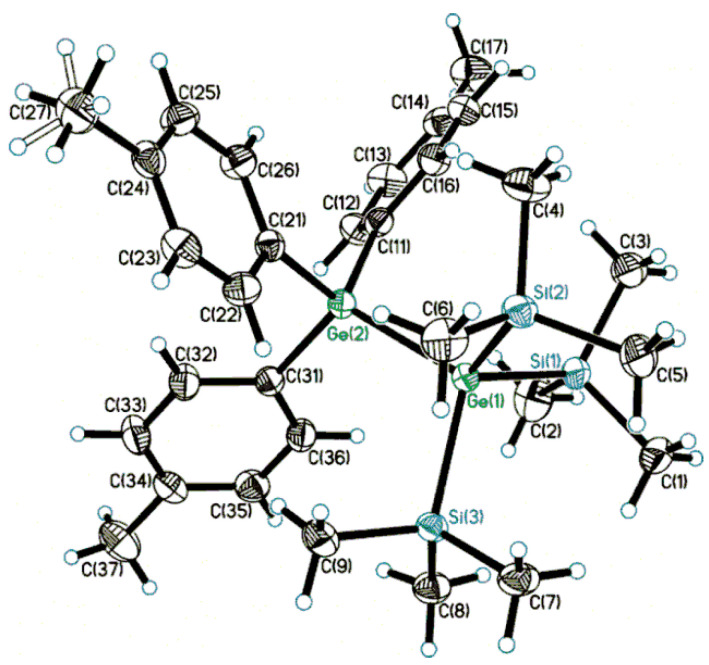
Molecular structure of compound (Me_3_Si)_3_Ge-Ge(C_6_H_4_Me-*p*)_3_ (**1**). Displacement ellipsoids are shown at a 50% probability level. Minor component of disordered Me group is drawn by open lines. Selected bond lengths (Å) and bond angles (deg): Ge(1)-Ge(2) 2.4393(2), Ge(1)-Si_av_ 2.3864(4), Ge(2)-C_av_ 1.9601(14); Si-Ge(1)-Si_av_ 107.973(16), Si-Ge(1)-Ge(2)_av_ 110.931(12), C-Ge(2)-C_av_ 107.68(6), C-Ge(2)-Ge(1)_av_ 111.21(4).

**Figure 2:**
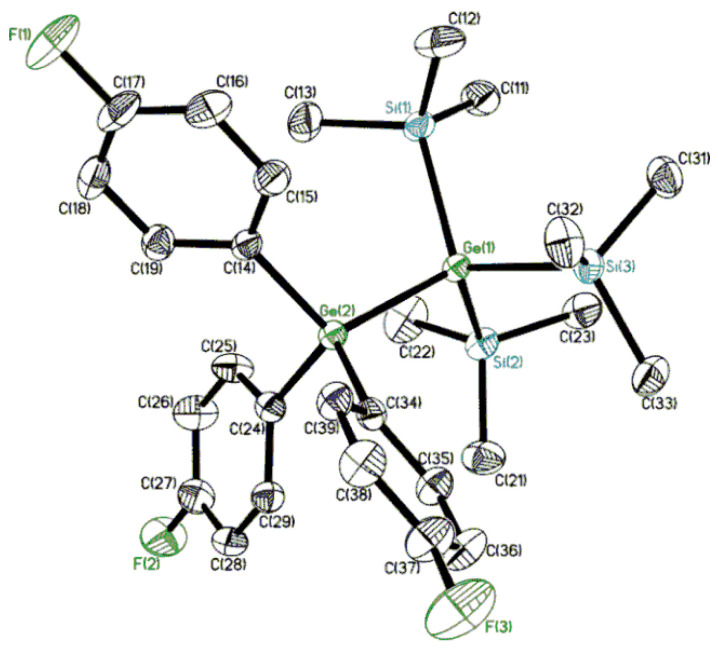
Molecular structure of compound (Me_3_Si)_3_Ge-Ge(C_6_H_4_F-*p*)_3_ (**2**). Only one independent molecule is drawn. Displacement ellipsoids are shown at a 50% probability level. Hydrogen atoms are omitted for clarity. Selected bond lengths (Å) and bond angles (deg): Ge(1)-Ge(2) 2.4431(3), Ge(1)-Si_av_ 2.3908(5), Ge(2)-C_av_ 1.9625(16); Si-Ge(1)-Si_av_ 109.350(18), Si-Ge(1)-Ge(2)_av_ 109.590(14), C-Ge(2)-C_av_ 107.49(7), C-Ge(2)-Ge(1)_av_ 111.39(5).

The molecular structures of molecular oligoorganogermanes **1** and **2** were studied in a crystal by X-ray diffraction analysis (Figure 1 and Figure 2; Supplementary Materials, Table S1). Interestingly, the molecular structures of compounds with the Si_3_Ge-GeC_3_ framework have not been studied previously.

The structural parameters of **1** and **2** are similar; an insignificant elongation of bond lengths is observed only at transition to **2**, containing electron-withdrawing substituents. This structural feature has been observed in such donor–acceptor oligoorganogermanes earlier [21]. The key Ge-Ge bond length is typical of digermanes (2.4393(2), 2,4431(3) Å vs. 2.393(3) [Ph_2_(O_2_CCCl_3_)Ge-Ge(O_2_CCCl_3_)Ph_2_] [32]–2.4787(7) [(Me_3_Si)_3_Ge-Ge(SiMe_3_)_3_] [22] Å; cf. 2.419(1) Å in (*p*-MeC_6_H_4_)_3_Ge-Ge(C_6_H_4_Me-*p*)_3_ [33] and 2.4209(8) Å in (*p*-FC_6_H_4_)_3_Ge-Ge(C_6_H_4_F-*p*)_3_ [34]), and the presence of sterically bulk hypergermyl, Hge [35,36], Ge(SiMe_3_)_3_ groups affects the elongation of the bond parameters. The Ge-Si bond lengths (average values, 2.3864(4) and 2.3908(5) Å, respectively) are within the normal range of 2.38–2.41 Å [22,37].

Germanium atoms in **1** and **2** adopt a slightly distorted tetrahedral geometry, T-4 (angle values vary within the range of 107°–112°). Tetrahedral τ_4_ parameters, τ_4_ = (360°-α-β)/141° (α, β are the largest bond angles) [38], are varied in the 0.96–0.99 range for Ge atoms in **1** and **2** (τ_4_ = 1 for an ideal T-4 geometry). The conformations of both molecules along the Ge-Ge bond (average Si-Ge-Ge-C torsions, 92.83(4)/27.17(4)° and 100.61(5)/19.39(5)°, respectively) can be described as distorted staggered (*ortho-*, *O-/cisoid-*, *C-* in terms of West [39]), which is noticeably different from the evident ideal 60° case (*C_3v_* symmetry). At the same time, in mixed (Me_3_Si)_3_Ge-GeAr_3_ the torsion distortion is more significant than in the parent compound (Me_3_Si)_3_Ge-Ge(SiMe_3_)_3_ (average Si-Ge-Ge-Si torsions, 76.82/43.18°) or (*p*-FC_6_H_4_)_3_Ge-Ge(C_6_H_4_F-*p*)_3_ (average Si-Ge-Ge-C torsions, 60.7(8)°). Thus, we can conclude that the introduction of sterically voluminous groups (such as trimethylsilyl instead of aryl) into catenated germanes affects the conformations more substantially (cf. the electron inducing eclipsed conformation in donor–acceptor digermanes [21]). Such torsion changes can even be accompanied by Ge-Ge bond length elongation.

Interestingly, the crystal of oligoorganogermane **2** is isostructural to Ph_3_Al*As(SiMe_3_)_3_ [40], indicating a similarity between crystals of different main group elements (Ge vs. Al, As).

### 2.4. DFT Calculations

**Scheme 3:**
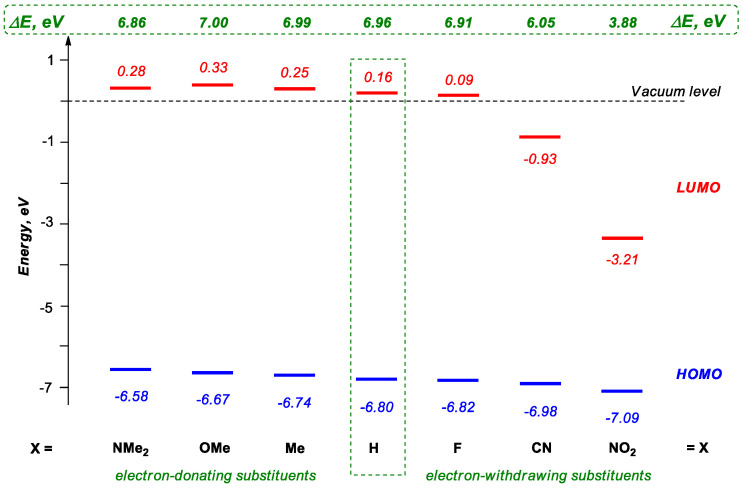
Schematic representation of HOMO, LUMO levels and HOMO/LUMO gap (ΔE) in model digermanes (Me_3_Si)_3_Ge-Ge(C_6_H_4_X-*p*)_3_.

We performed DFT calculations to clarify the properties of investigated digermanes using model compounds, (Me_3_Si)_3_Ge-Ge(C_6_H_4_X-*p*)_3_, in which the electronic properties of the Ar group were changed. In addition to the parent compound (Me_3_Si)_3_Ge-GePh_3_ (X = H), we studied electron-donating (X = Me, OMe, NMe_2_) and electron-withdrawing (X = F, CN, NO_2_) substituents in the aromatic ring. The levels of the HOMO and LUMO (lowest unoccupied molecular orbital) as well as the frontier orbital energy gap (ΔE) are presented in Scheme 3.

It should be noted one more time that before this work only (Me_3_Si)_3_Ge-GePh_3_ (X = H) [27,41] had been synthesized and investigated.

We found that the change of the electron properties of the Ar group in (Me_3_Si)_3_Ge-Ge(C_6_H_4_X-*p*)_3_ determined the energy of frontier orbitals. At the introduction of electron *donating* groups, the HOMO level was destabilized (increased in energy); this has been observed earlier by Weinert et al. [20] for related derivatives. At the same time, the LUMO level was also destabilized, but the general change of the HOMO/LUMO gap was insignificant. The narrowing of the energy gap in (Me_3_Si)_3_Ge-Ge(C_6_H_4_X-*p*)_3_ was observed only for strong donors (X = NMe_2_). A more complex situation was observed for electron-*withdrawing* groups; it depended strongly on the type of X. The typical feature in this case was the stabilization of the HOMO level; a stronger acceptor led to a greater energy decrease. Furthermore, a stronger *acceptor* led to a more significant LUMO stabilization, especially in comparison with the HOMO level change. In other words, in the case of electron-withdrawing substituents X, the decrease in the ΔE associated first of all with the stabilization of LUMO, leading to a remarkable decrease in its energy level. All this indicates that the introduction of strong withdrawing substituents should lead to a more significant HOMO/LUMO gap decrease. The most striking data were obtained for a yet unknown (calculated but not yet synthesized) NO_2_ derivative, which challenges the need for such compounds.

**Figure 3:**
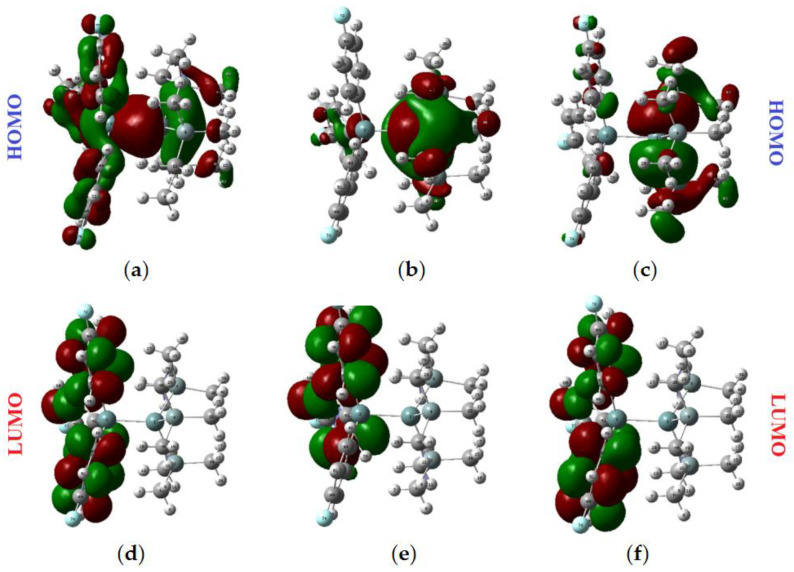
Graphical representation of the frontier orbitals of (Me_3_Si)_3_Ge-Ge(C_6_H_4_F-*p*)_3_ (**2**): (**a**) HOMO; (**b**) HOMO-1; (**c**) HOMO-2; (**d**) LUMO; (**e**) LUMO+1; (**f**) LUMO+2.

The distribution of electron density in HOMO and LUMO orbitals in (Me_3_Si)_3_Ge-Ge(C_6_H_4_F-*p*)_3_ (**2**) is presented in Figure 3; the data for (Me_3_Si)_3_Ge-GePh_3_ are given in Supplementary Materials (Figure S14). For these digermanes, (Me_3_Si)_3_Ge-GeAr_3_, the HOMO was distributed on the Ge-Ge bond with the inclusion of an Ar group, but to a lesser extent, which is typical of aryl oligoorganogermanes. The more stabilized HOMO-1 and HOMO-2 are localized on Ge-Si bonds. As is typical of arylgermanes, the LUMO and the higher-energy LUMO+1 and LUMO+2 were concentrated on aryl substituents. These data indicate that UV/vis absorption (Table 1) corresponds to σ,π-transitions (Ge-Ge-Si to Ar), making the absorbance bands (see below), to some extent, less intensive in comparison with related compounds, where the HOMO and LUMO are on E-E bonds (σ-transitions, as in fully alkylated or alkylsilylated oligogermanes).

Theoretical analysis of UV/vis absorption spectral data for (Me_3_Si)_3_Ge-GeAr_3_ (Table 1) shows a good correlation with the experiment (see Table 2 below).

### 2.5. UV/Vis Absorption

Absorption data for compounds **1**–**3** and related derivatives are given in Table 2. In general, investigation of UV/vis absorption is highly important for studies of catenated main group element derivatives and can be regarded as an estimation of conjugation in them.

These data clearly show that in digermanes the absorption maximum (and therefore the HOMO/LUMO gap) depends strongly on σ,π-conjugation (involvement of Ar groups in conjugation with Ge and Ge-Ge frameworks); the presence of aromatic substituents results in a red shift. At the same time, for (Me_3_Si)_3_Ge-GeAr_3_, the electron properties of Ar affect UV/vis absorption, which is consistent with the DFT data. Thus, compounds with strong acceptor groups are characterized by a greater bathochromic shift; introduction of donating groups also results in red changes in (C_6_F_5_ > *p*-MeC_6_H_4_ > Ph > *p*-FC_6_H_4_) spectra. Furthermore, the Ge-Si conjugation also affects the shift of the absorption band. Besides, linear conjugation is more effective than the branched one (Si-Si-Ge vs. Si_3_-Ge).

## 3. Materials and Methods

### 3.1. Experimental Details

All manipulations were performed under a dry and oxygen-free argon atmosphere using the standard Schlenk techniques. The ^1^H (400.130 MHz), ^13^C (100.613 MHz), ^19^F (376.498 MHz), and ^29^Si (79.495 MHz) NMR spectra were recorded on a Bruker 400 or Agilent 400MR spectrometer at 298 K. Chemical shifts are given in ppm relative to internal Me_4_Si (^1^H, ^13^C, and ^29^Si NMR spectra) or external CFCl_3_ (^19^F spectra). Elemental analyses were carried out at the Microanalytical Laboratory, Chemistry Department, M.V. Lomonosov Moscow State University, using a Heraeus Vario Elementar instrument or at the Laboratory of Microanalysis, N.D. Zelinsky Institute of Organic Chemistry RAS on a PerkinElmer 2400 Series II CHN Elemental Analyzer. Matrix-assisted laser-desorption/ionization time-of-flight mass spectrometry (MALDI-TOF-MS) analyses were performed on a Microflex (Bruker Daltonics) time-of-flight mass spectrometer; the spectra were recorded in the positive linear mode. UV/visible spectra were obtained using a Thermo Scientific Evolution 300 double-beam spectrophotometer with a 0.10 cm cuvette.

Solvents were dried by standard methods and distilled prior to use. Tetrahydrofuran was stored under solid KOH and then distilled over sodium/benzophenone; *n*-hexane was refluxed and distilled over sodium. CDCl_3_ was refluxed and distilled over CaH_2_ under argon atmosphere. 

Starting materials, (Me_3_Si)_4_Ge [45], (*p*-MeC_6_H_4_)_3_GeCl [42,46], (*p*-FC_6_H_4_)_3_GeCl [34], and (C_6_F_5_)_3_GeCl [21], were obtained via previously reported procedures. *t*-BuOK and other reagents were used as supplied (Aldrich). 

### 3.2. X-ray Crystallography

Crystal data, data collection, structure solution and refinement parameters for **1** and **2** are given in Table S1 (Supplementary Materials). Experimental intensities were measured on a Bruker SMART APEX II diffractometer (graphite monochromatized Mo-Kα radiation, λ = 0.71073 Å) using the *ω* scan mode. The structures were solved by direct methods and refined by full-matrix least-squares on *F*^2^ (*SHELXTL*) with anisotropic thermal parameters for all non-hydrogen atoms. All hydrogen atoms were placed in calculated positions and refined using a riding model. In **1**, one of the methyl groups was found to be rotationally disordered. X-ray diffraction studies were performed at the Centre of Shared Equipment of IGIC RAS. Crystallographic data were deposited with the Cambridge Crystallographic Data Centre as supplementary publications nos. CCDC-2145489 and 2145490. 

### 3.3. DFT Calculations

The calculations were performed with full geometry optimization and used the GAUSSIAN’09 program package [47]. The absence of imaginary vibration frequencies confirmed the stationary character of the structures. The hybrid *meta* exchange-correlation functional called M06-2X was used. It is a high-nonlocality functional with double the amount of nonlocal exchange (2X), parameterized only for nonmetals [48]. We used the time-dependent density functional computations [6–31 G (d, p) basis set], as implemented by Gaussian 09, which were utilized to explore the excited manifold and to compute the possible electronic transitions. The molecular orbitals and UV/visible spectra were constructed using the GaussView program. The UV spectra were calculated in an approximation of the polarizable continuum model in dichloromethane [49].

### 3.4. Synthesis

Synthesis of Ar_3_Ge-Ge(SiMe_3_)_3_. General procedure. Step 1, synthesis of [(Me_3_Si)_3_Ge*THF] in situ. The procedure of Marschner et al. was used [30]. Solid *t*-BuOK (0.1540 g, 1.37 mmol) was added to a solution of (Me_3_Si)_4_Ge (0.5000 g, 1.37 mmol) in THF (20 mL). The mixture obtained was stirred for 5 h. The solution of [(Me_3_Si)_3_Ge*THF] in THF was used further without additional purification.

**Step 2, synthesis of Ar_3_Ge-Ge(SiMe_3_)_3_.** At −78 °C, the solution of [(Me_3_Si)_3_Ge*THF] in THF, obtained as stated above in *Step 1*, was added dropwise to a solution of Ar_3_GeCl (1.00 eq., 1.37 mmol) in THF (20 mL). The mixture obtained was stirred at the same temperature for 2 h, slowly warmed to room temperature and stirred overnight. Then, all volatile materials were removed under reduced pressure, and the residue was purified by passing through a pad of SiO_2_ using petroleum ether as an eluent. After evaporation, the solid obtained was recrystallized from a minimal amount of *n*-hexane to give Ar_3_Ge-Ge(SiMe_3_)_3_.

*1,1,1-Tris(p-tolyl)-2,2,2-tris(trimethylsilyl) digermane, (p-MeC_6_H_4_)_3_Ge-Ge(SiMe_3_)_3_* (**1**). White powder. Yield: 0.6023 g (69%).

^1^H NMR (400.130 MHz, CDCl_3_): δ 0.14 (s, 27H, 3SiMe_3_); 2.34 (s, 9H, 3 *Me*C_6_H_4_-*p*); 7.12 (d, ^3^*J_H-H_* = 7.7 Hz, 6H, 3 *meta-*(*p*-C_6_H_4_)), 7.33 (d, ^3^*J_H-H_* = 7.7 Hz, 6H, 3 *ortho-*(*p*-C_6_H_4_)).

^13^C{^1^H} NMR (100.613 MHz, CDCl_3_): δ 3.30 (^1^*J_13C-29Si_* = 45.4 Hz, SiMe_3_); 21.43 (*Me*C_6_H_4_-*p*); 128.65 (*meta-*C_6_H_4_), 135.45 (*ortho-*C_6_H_4_), 136.86 (*ipso-*C_6_H_4_), 137.76 (*para*-C_6_H_4_).

^29^Si{^1^H} NMR (79.495 MHz, CDCl_3_): δ –4.48 (SiMe_3_).

MALDI-TOF MS: m/z 638 [M]^+^.

UV/visible absorption (*n*-hexane, *λ*_max_ in nm (*ε* in M^−1^ cm^−1^)): 239 (3.1 × 10^4^).

Anal. Calcd for C_30_H_48_Ge_2_Si_3_ (M_w_ 638.2386): C, 56.46; H, 7.58%. Found: C, 56.22; H, 7.38%.

Single crystals of compound **1** were obtained after recrystallization from *n*-octane at −30 °C.

*1,1,1-Tris(p-fluorophenyl)-2,2,2-tris(trimethylsilyl) digermane, (p-FC_6_H_4_)_3_Ge-Ge(SiMe_3_)_3_* (**2**). White powder. Yield: 0.6226 g (70%).

^1^H NMR (400.130 MHz, CDCl_3_): δ 0.14 (s, 27H, 3SiMe_3_); 7.05 (pt, *J_H-H_* = 8.6 Hz, 6H, 3 *meta*-(*p*-C_6_H_4_)), 7.36 (dd, ^3^*J_H-H_* = 8.4 Hz, ^3^*J_1H-19F_* = 6.3 Hz, 6H, 3 *ortho*-(*p*-C_6_H_4_)).

^13^C{^1^H} NMR (100.613 MHz, CDCl_3_): δ 3.24 (^1^*J_13C-29Si_* = 45.4 Hz, SiMe_3_); 115.30 (d, ^2^*J_13C-19F_* = 19.8 Hz, *meta*-C_6_H_4_), 135.03 (d, ^4^*J_13C-19F_* = 3.7 Hz, *ipso-*C_6_H_4_), 136.95 (d, ^3^*J_13C-19F_* = 7.3 Hz, *ortho*-C_6_H_4_), 163.38 (d, ^1^*J_13C-19F_* = 248.1 Hz, *para*-C_6_H_4_).

^19^F NMR (376.498 MHz, CDCl_3_): *δ* −112.83 (1F).

^29^Si{^1^H} NMR (79.495 MHz, CDCl_3_): δ –4.30 (SiMe_3_).

MALDI-TOF MS: m/z 650 [M]^+^.

UV/visible absorption (*n*-hexane, *λ*_max_ in nm (*ε* in M^−1^ cm^−1^)): 230 (3.3×10^4^).

Anal. Calcd for C_27_H_39_F_3_Ge_2_Si_3_ (M_w_ 650.1303): C, 49.88; H, 6.05%. Found: C, 50.12; H, 5.92%.

Single crystals of compound **2** were obtained after recrystallization from *n*-octane at −30 °C.

*1,1,1-Tris(p-fluorophenyl)-2,2,2-tris(trimethylsilyl) digermane, (C_6_F_5_)_3_Ge-Ge(SiMe_3_)_3_* (**3**). The crude product was purified by column chromatography (SiO_2_, petroleum ether, R_f_ 0.2). White powder. Yield: 0.1124 g (10%).

^1^H NMR (400.130 MHz, CDCl_3_): δ 0.23 (s, 27H, 3SiMe_3_).

^13^C{^1^H} NMR (100.613 MHz, CDCl_3_): δ 3.28 (^1^*J_13C-29Si_* = 44.3 Hz, SiMe_3_); 135.93–136.35 (m), 138.74–138.85 (m), 142.20–142.59 (m), 146.95–147.13 (m), 145.51–149.56 (m) (C_6_F_5_). Several signals of carbons of C_6_F_5_ groups were not found due to low intensity and high value of nuclear coupling.

^19^F NMR (376.498 MHz, CDCl_3_): *δ* −158.91 − (−158.80) (m, 2F), −147.72 − (−147.33) (m, 1F), −126.49 − (−126.46) (m, 2F).

^29^Si{^1^H} NMR (79.495 MHz, CDCl_3_): δ –5.33 (SiMe_3_).

MALDI-TOF MS: m/z 866 [M]^+^.

UV/visible absorption (*n*-hexane, *λ*_max_ in nm (*ε* in M^−1^ cm^−1^)): 242 (3.1 × 10^4^).

Anal. Calcd for C_27_H_27_F_15_Ge_2_Si_3_ (M_w_ 866.0158): C, 37.45; H, 3.14%. Found: C, 37.08; H, 3.12%.

## 4. Conclusions

The results reported in the present work can be regarded as significant advances in main group metal chemistry. We showed that arylated and trimethylsilylated oligoorganogermanes could be easily synthesized by the interaction between aryl halogermanes and germyl potassium salts. Effective σ,π-conjugation between different structural fragments, Ar-Ge and Si-Ge-Ge, resulting in a bathochromic shift of absorption bands, was observed; the conjugation was explained by a HOMO/LUMO gap decrease. The conformational behavior of catenated germanes was determined by the steric size of the substituents; the effect of substituents’ size on bond lengths was less significant. The influence of chemical (number and type of E atoms in conjugation, electronic effects of substituents) and structural (conformational behavior) factors on the properties of oligogermanes makes them an attractive subject for further investigation by luminescence, conductive, thermal, and electrochemical methods. The intriguing properties of a series of substituted derivatives, found by DFT calculations, especially for a number of electron-withdrawing (X = NO_2_) compounds, will stimulate their synthesis.

## Figures and Tables

**Table 1:** Data of DFT calculations for compounds of the type of (Me_3_Si)_3_Ge-GeAr_3._

Compound	λ_max_ (Calcd.), nm	Oscillator Strength, f	Transition
(Me_3_Si)_3_Ge-GePh_3_	228	0.35 (0.15)	HOMO–LUMO+1
(Me_3_Si)_3_Ge-Ge(C_6_H_4_Me-*p*)_3_ (**1**)	239	0.46 (0.11)	HOMO-2–LUMO
(Me_3_Si)_3_Ge-Ge(C_6_H_4_OMe-*p*)_3_	235	0.57 (0.22)	HOMO-2–LUMO
(Me_3_Si)_3_Ge-Ge(C_6_H_4_NMe_2_-*p*)_3_	244	0.28 (0.58)	HOMO-2–LUMO
(Me_3_Si)_3_Ge-Ge(C_6_H_4_F-*p*)_3_ (**2**)	233	0.28 (0.27)	HOMO-2–LUMO+1
(Me_3_Si)_3_Ge-Ge(C_6_H_4_CN-*p*)_3_	261	0.42 (0.22)	HOMO-2–LUMO+1
(Me_3_Si)_3_Ge-Ge(C_6_H_4_NO_2_-*p*)_3_	518	0.18 (0.01)	HOMO-2–LUMO+2

**Table 2:** Comparison of absorption maxima in UV/visible spectra for **1**–**3** and related compounds.

Compound	*λ_max_*, nm (*ε* × 10^−4^, M^−1^ cm^−1^)	Solvent	Reference
Me_3_Ge-Ge(C_6_H_4_Me-*p*)_3_	234 (3.7)	CH_2_Cl_2_	[27]
(*p*-MeC_6_H_4_)_3_Ge-Ge(C_6_H_4_Me-*p*)_3_	241 (1.8)	CH_2_Cl_2_	[42]
(*p*-MeC_6_H_4_)_3_Ge-Ge(C_6_F_5_)_3_	234 (4.6)	CH_2_Cl_2_	[21]
(*p*-MeC_6_H_4_)_3_Ge-GePh_3_	240	CH_2_Cl_2_	[42]
(Me_3_Si)_3_Ge-Ge(SiMe_3_)_3_	209 (7.8)	*n*-pentane	[22]
(Me_3_Si)_3_Ge-GeCl_3_	231 (1.4)	*n*-hexane	[21]
(Me_3_Si)_3_Ge-GePh_3_	234 (2.7)	*n*-hexane	[27]
Me_3_Si-Ge(C_6_H_4_Me-*p*)_3_	230 (3.3)	*n*-hexane	[27]
Me_3_Si-Ge(C_6_H_4_Me-*p*)_3_	231 (1.8)	CH_2_Cl_2_	[27]
(*t*-Bu)Me_2_Si-Ge(C_6_H_4_Me-*p*)_3_	233 (3.2)	*n*-hexane	[43]
(*t*-Bu)Me_2_Si-Ge(C_6_H_4_Me-*p*)_3_	235 (4.5)	CH_2_Cl_2_	[43]
[Me_3_Si]_2_-GePh_2_	238 (1.5)	*n*-pentane	[44]
Me_3_Si-Me_2_Si-Ge(C_6_H_4_Me-*p*)_3_	241 (2.5)	CH_2_Cl_2_	[16]
(Me_3_Si)_3_Ge-Ge(C_6_H_4_Me-*p*)_3_ (1)	239 (3.1)	*n*-hexane	this work
(Me_3_Si)_3_Ge-Ge(C_6_H_4_F-*p*)_3_ (2)	230 (3.3)	*n*-hexane	this work
(Me_3_Si)_3_Ge-Ge(C_6_F_5_)_3_ (3)	242 (3.1)	*n*-hexane	this work

## Data Availability

Not applicable.

## Supplementary Materials

The following supporting information can be downloaded at: https://www.mdpi.com/article/10.3390/molecules27072147/s1, Figures S1–S11 (NMR spectra of the compounds obtained), Figures S12–S14 (UV/vis spectra of the compounds obtained), Table S1 (crystallographic data), Figure S14 (DFT data): Supporting_Information_Molecules.

## Sample Availability

Samples of the compounds are available from the authors.
