# Supplementary material for: Oligoorganogermanes: Interplay between Aryl and Trimethylsilyl Substituents
Source: Molecules. 2022 Mar 26;27(7):2147. doi: 10.3390/molecules27072147 (PMC9000802; doi:10.3390/molecules27072147)
Supplement: Supplementary file 1 [file molecules-27-02147-s001.zip › Biography1.pdf]

Author Biography

Dr. Habil. Kirill V. Zaitsev works as a Leading Researcher, Chemistry Department, Lomonosov Moscow State University (MSU). As Leading Researcher he part-time worked at the institutes of the Russian Academy of Sciences (N.D. Zelinsky Institute of Organic Chemistry, A.N. Nesmeyanov Institute of Organoelement Compounds, A.V. Topchiev Institute of Petrochemical Synthesis). He educated (2003, M. Sc.) in Chemistry Department, MSU; in 2006 defended his Ph.D. thesis; in 2020 he habilitated (defended his Dr. Habil.). Dr. Habil. K.V. Zaitsev worked as Researcher in University of Marburg (Germany), as Invited Lecturer in Graz University of Technology (Austria), as Invited Professor in Shandong University (China). He works in the field of organometallic chemistry, organic synthesis, asymmetric synthesis, polymer chemistry, chemistry of natural compounds. Dr. Habil. Zaitsev is a member of International Advisory Board of the International Conferences on the Coordination and Organometallic Chemistry of Germanium, Tin and Lead (ICCOC-GTL).
