# Supplementary material for: Oligoorganogermanes: Interplay between Aryl and Trimethylsilyl Substituents
Source: Molecules. 2022 Mar 26;27(7):2147. doi: 10.3390/molecules27072147 (PMC9000802; doi:10.3390/molecules27072147)
Supplement: Supplementary file 1 [file molecules-27-02147-s001.zip › Supporting_Information_Molecules.pdf]

Supporting Information

for

Oligoorganogermanes: interplay between aryl and trimethylsilyl substituents

Kirill V. Zaitsev[a, b, *] Yurii F. Oprunenko,[a] Oleg Kh. Poleshchuk,[c, d] Andrei V. Churakov [e]

[a] *Department of Chemistry, Moscow State University, Leninskie Gory 1, 3, Moscow 119991, Russia*

[b] *A.V. Topchiev Institute of Petrochemical Synthesis, Russian Academy of Sciences, Leninskii Pr., 29, Moscow, 119991, Russia*

[c] *National Research Tomsk State University, Lenin Av., 30, Tomsk 634050, Russia*

[d] *Tomsk State Pedagogical University,Kievskaya Str., 60, Tomsk 634061, Russia*

[e] *N.S. Kurnakov Institute of General and Inorganic Chemistry, Russian Academy of Sciences, Leninsky Pr. 31, Moscow 117901, Russia*

** Corresponding author:*

E-mail address: zaitsev@org.chem.msu.ru; fax: +7(495)939 0067; phone: +7(495)939 3887

Postal address: Department of Chemistry, M.V. Lomonosov Moscow State University, Leninskie Gory 1, Bldg 3, GSP-1, Moscow 119991, Russia

Table of Contents

NMR Spectra of the Compounds Obtained

Fig. S1. ^{1}H NMR spectrum of $(Me_3Si)_3$Ge-Ge$(C_6H_4Me$-*p*$)_3$ (**1**) ($CDCl_3$, RT)……………………………………..S3
Fig. S2. ^{13}C NMR spectrum of $(Me_3Si)_3$Ge-Ge$(C_6H_4Me$-*p*$)_3$ (**1**) ($CDCl_3$, RT)..…………………………………S3
Fig. S3. ^{29}Si NMR spectrum of $(Me_3Si)_3$Ge-Ge$(C_6H_4Me$-*p*$)_3$ (**1**) ($CDCl_3$, RT)..………………………………..S4
Fig. S4. ^{1}H NMR spectrum of $(Me_3Si)_3$Ge-Ge(*p*-$C_6H_4F)_3$ (**2**) ($CDCl_3$, RT)..……………………………………..S4
Fig. S5. ^{13}C NMR spectrum of $(Me_3Si)_3$Ge-Ge(*p*-$C_6H_4F)_3$ (**2**) ($CDCl_3$, RT)....…………………………………..S5
Fig. S6. ^{19}F NMR spectrum of $(Me_3Si)_3$Ge-Ge(*p*-$C_6H_4F)_3$ (**2**) ($CDCl_3$, RT)……………………………………..S5
Fig. S7. ^{29}Si NMR spectrum of $(Me_3Si)_3$Ge-Ge(*p*-$C_6H_4F)_3$ (**2**) ($CDCl_3$, RT)……………………………………..S6
Fig. S8. ^{1}H NMR spectrum of $(Me_3Si)_3$Ge-Ge$(C_6F_5)_3$ (**3**) ($CDCl_3$, RT)....……………………………………..S6
Fig. S9. ^{13}C NMR spectrum of $(Me_3Si)_3$Ge-Ge$(C_6F_5)_3$ (**3**) ($CDCl_3$, RT).....……………………………………...S7
Fig. S10. ^{19}F NMR spectrum of $(Me_3Si)_3$Ge-Ge$(C_6F_5)_3$ (**3**) ($CDCl_3$, RT) .....……………………………………S7

Fig. S11. ^{29}Si NMR spectrum of $(Me_3Si)_3Ge\text{-}Ge(C_6F_5)_3$ (**3**) ($CDCl_3$, RT) .....……………………………………S8

UV/vis Absorption Spectra of the Compounds Obtained

Fig. S12. UV/vis Absorption Spectrum of $(Me_3Si)_3Ge\text{-}Ge(C_6H_4Me\text{-}p)_3$ (**1**) (*n*-hexane, RT).……...……………S9
Fig. S13. UV/vis Absorption Spectrum of $(Me_3Si)_3Ge\text{-}Ge(p\text{-}C_6H_4F)_3$ (**2**) (*n*-hexane, RT)……………………..S10

Data of the Single Crystal X-ray Diffraction Analysis

Table S1. Crystallographic data for complex $(Me_3Si)_3Ge\text{-}Ge(C_6H_4Me\text{-}p)_3$ (**1**) and $(Me_3Si)_3Ge\text{-}Ge(p\text{-}C_6H_4F)_3$ (**2**).....….......…...……………………………………………………………S11

Data of DFT calculations

Fig. S14. Graphical representation of HOMO (**A**), HOMO-1 (**B**), HOMO-2 (**C**), LUMO (**D**), LUMO+1 (**E**), LUMO+2 (**F**) for compound $(Me_3Si)_3Ge\text{-}GePh_3$…….…..…..……………………………………S12

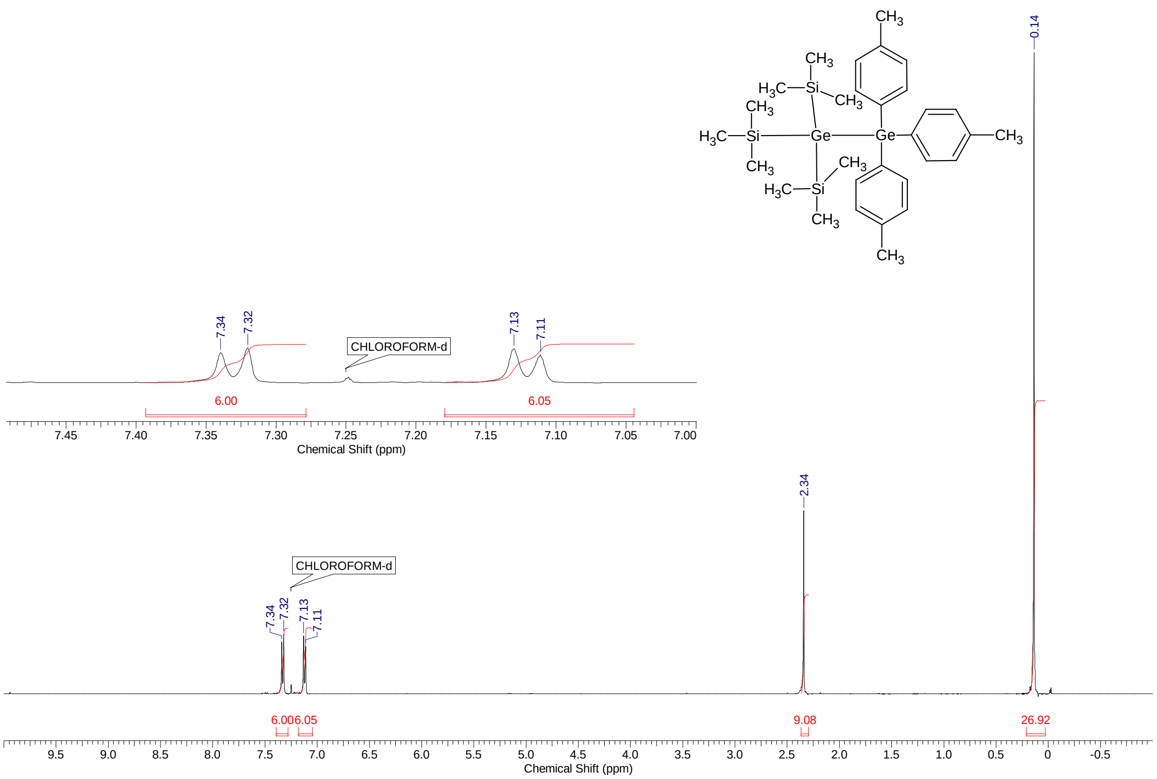

Fig. S1. ^{1}H NMR spectrum of $(Me_3Si)_3Ge\text{-}Ge(C_6H_4Me\text{-}p)_3$ (**1**) ($CDCl_3$, RT).

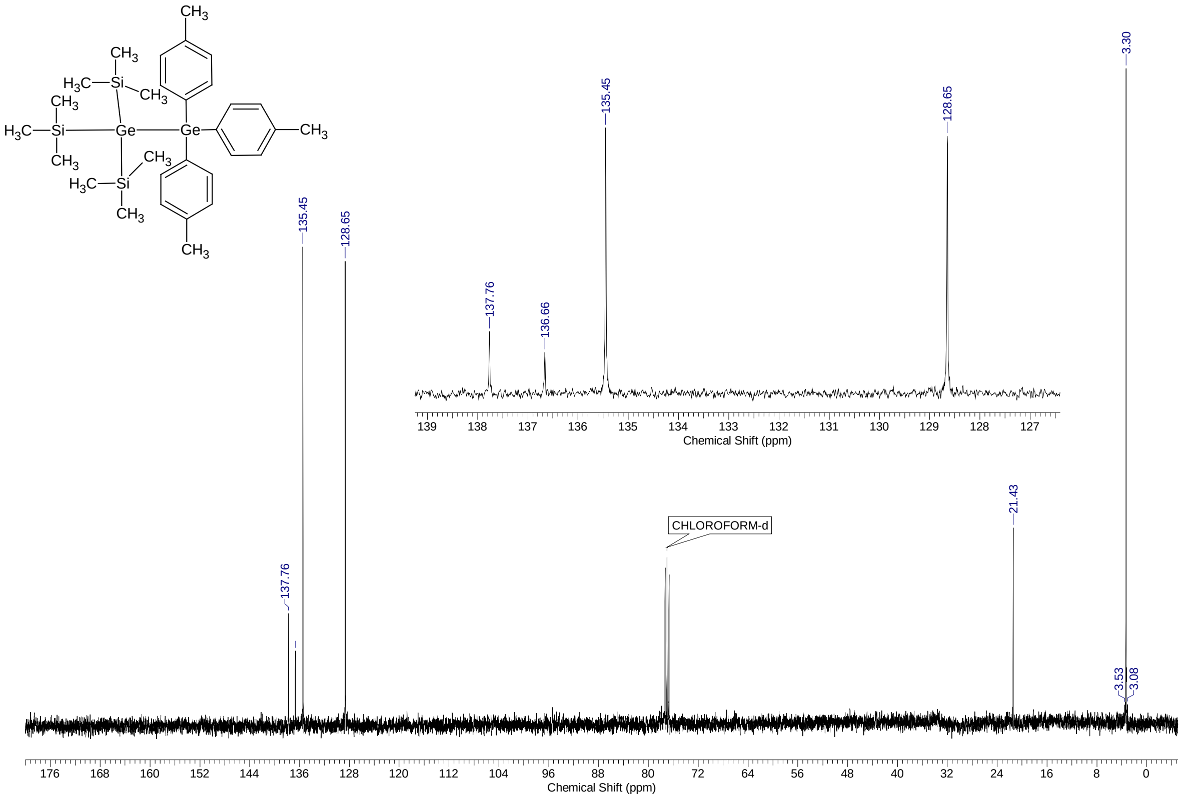

Fig. S2. ^{13}C NMR spectrum of $(Me_3Si)_3Ge\text{-}Ge(C_6H_4Me\text{-}p)_3$ (**1**) ($CDCl_3$, RT).

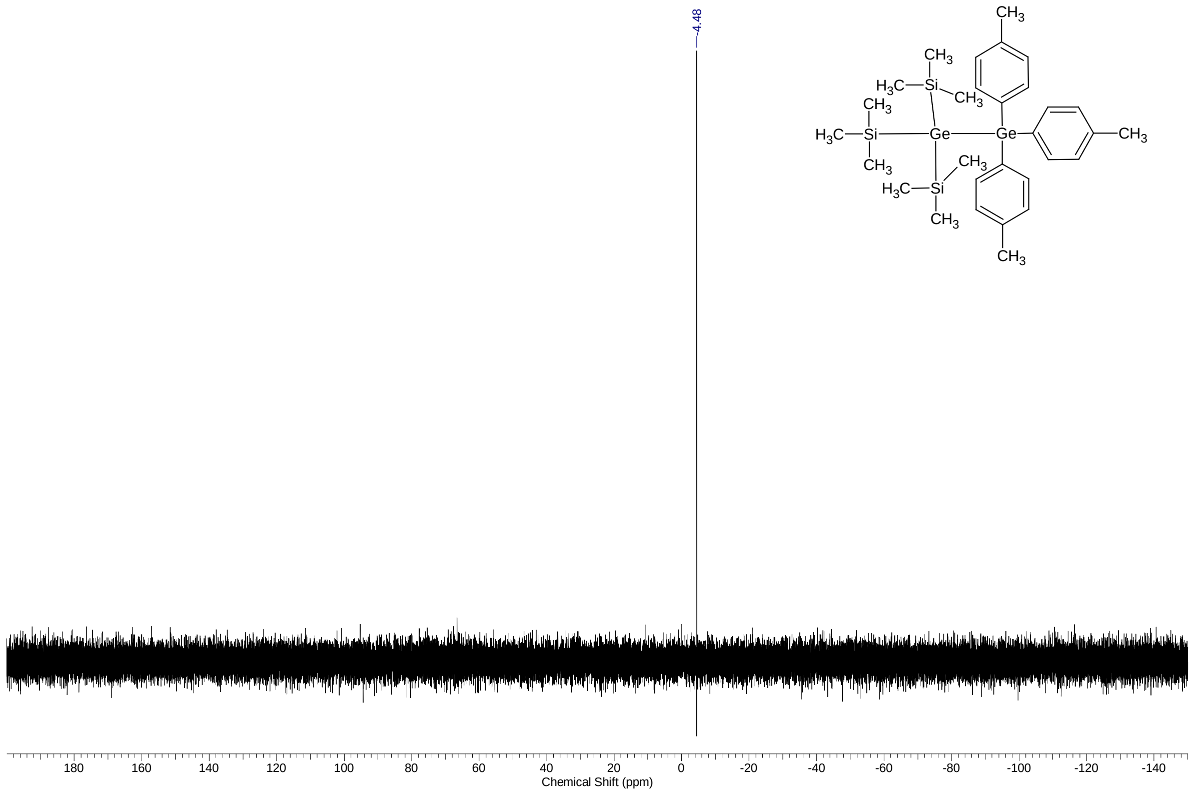

Fig. S3. ^{29}Si NMR spectrum of $(Me_3Si)_3Ge$-$Ge(C_6H_4Me$-$p)_3$ (**1**) ($CDCl_3$, RT).

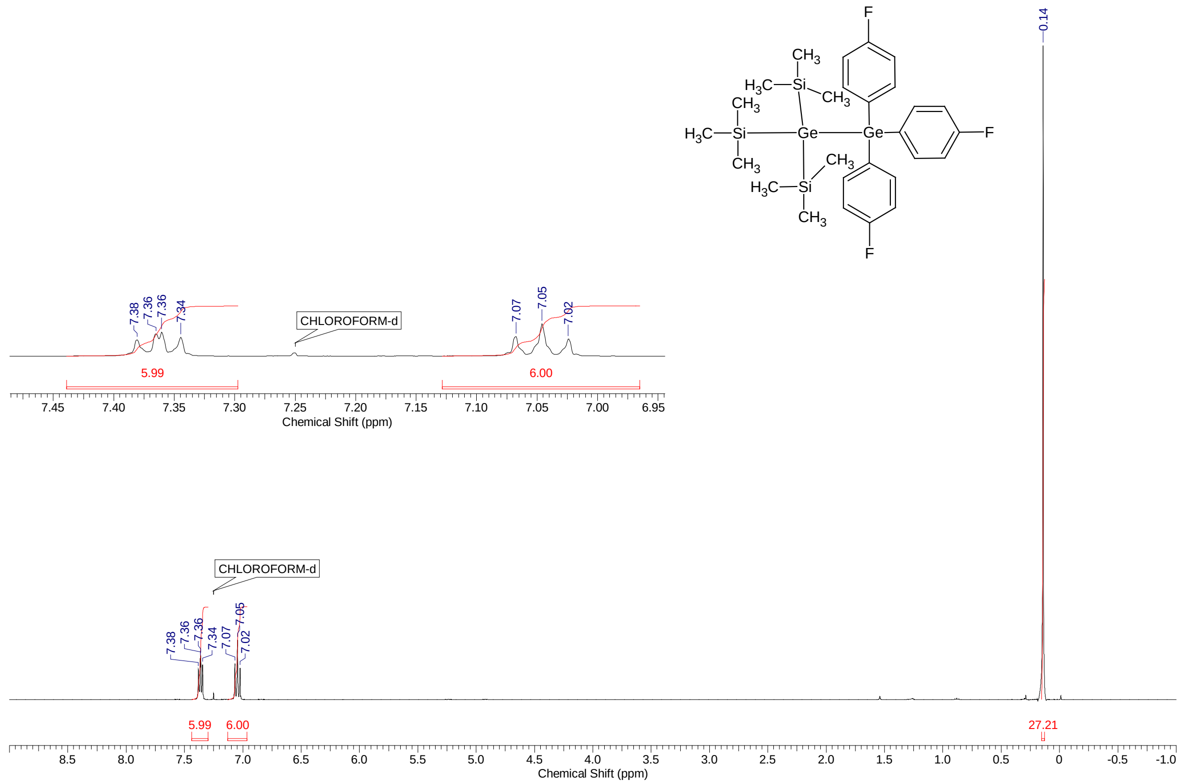

Fig. S4. ^{1}H NMR spectrum of $(Me_3Si)_3Ge$-$Ge(p$-$C_6H_4F)_3$ (**2**) ($CDCl_3$, RT).

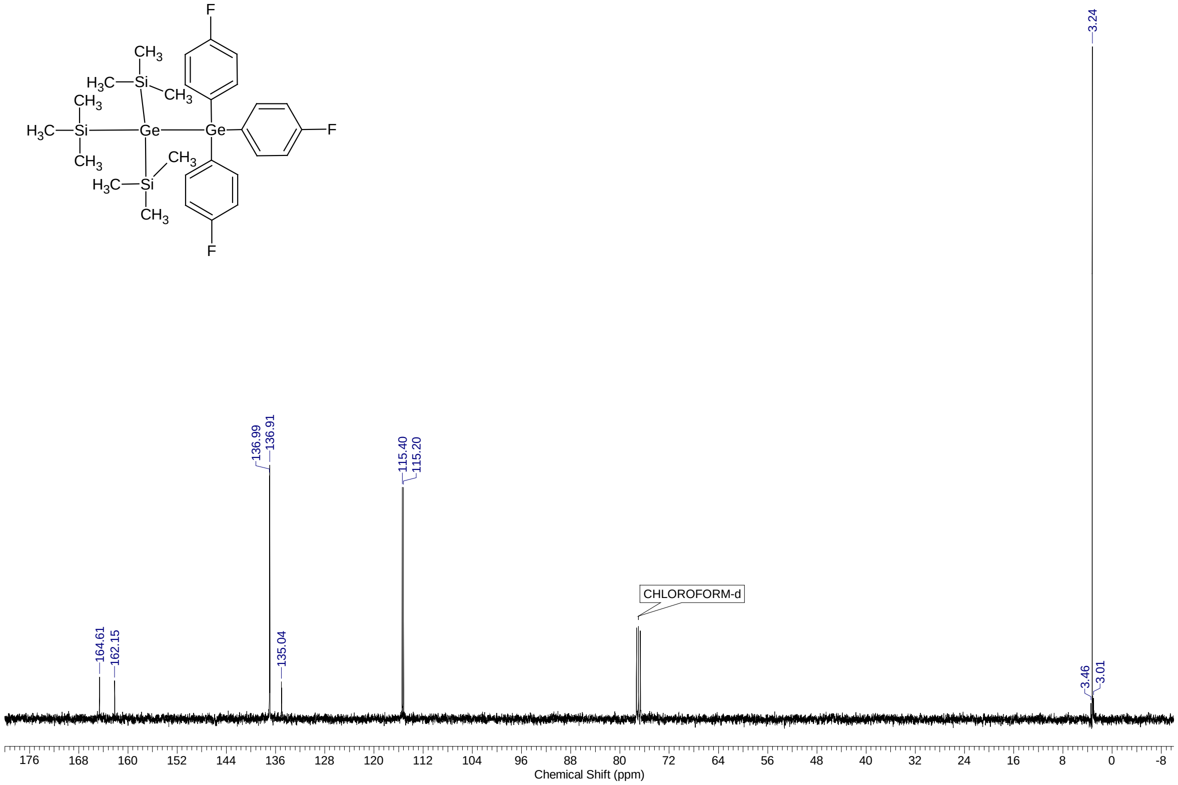

Fig. S5. ^{13}C NMR spectrum of $(Me_3Si)_3$Ge-Ge(p-$C_6H_4F)_3$ (**2**) ($CDCl_3$, RT).

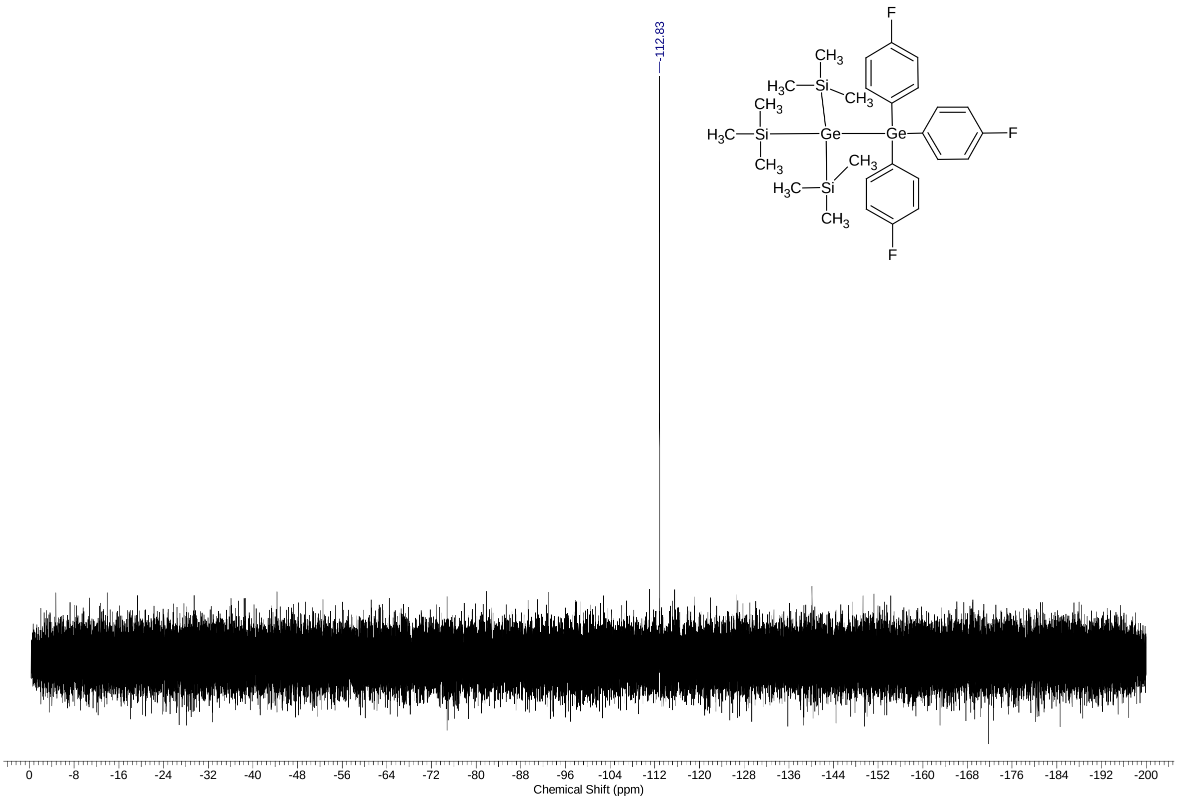

Fig. S6. ^{19}F NMR spectrum of $(Me_3Si)_3$Ge-Ge(p-$C_6H_4F)_3$ (**2**) ($CDCl_3$, RT).

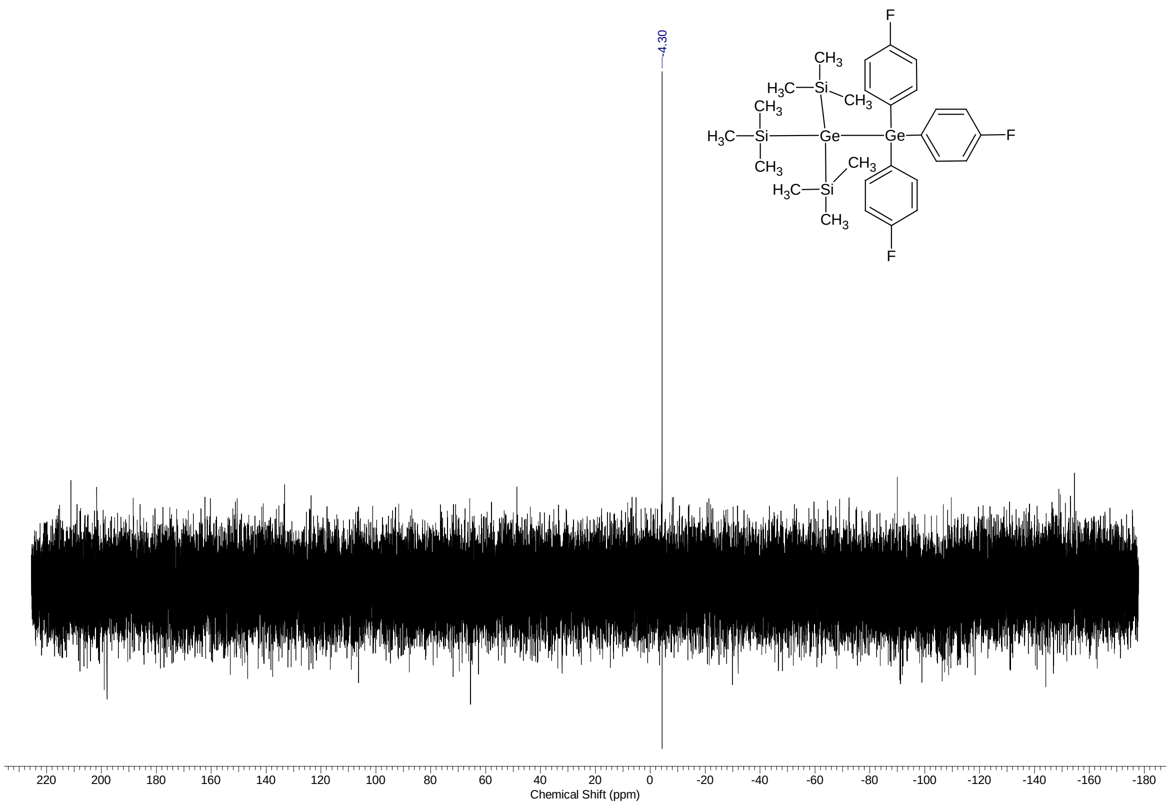

Fig. S7. ^{29}Si NMR spectrum of $(Me_3Si)_3Ge\text{-}Ge(p\text{-}C_6H_4F)_3$ (**2**) ($CDCl_3$, RT).

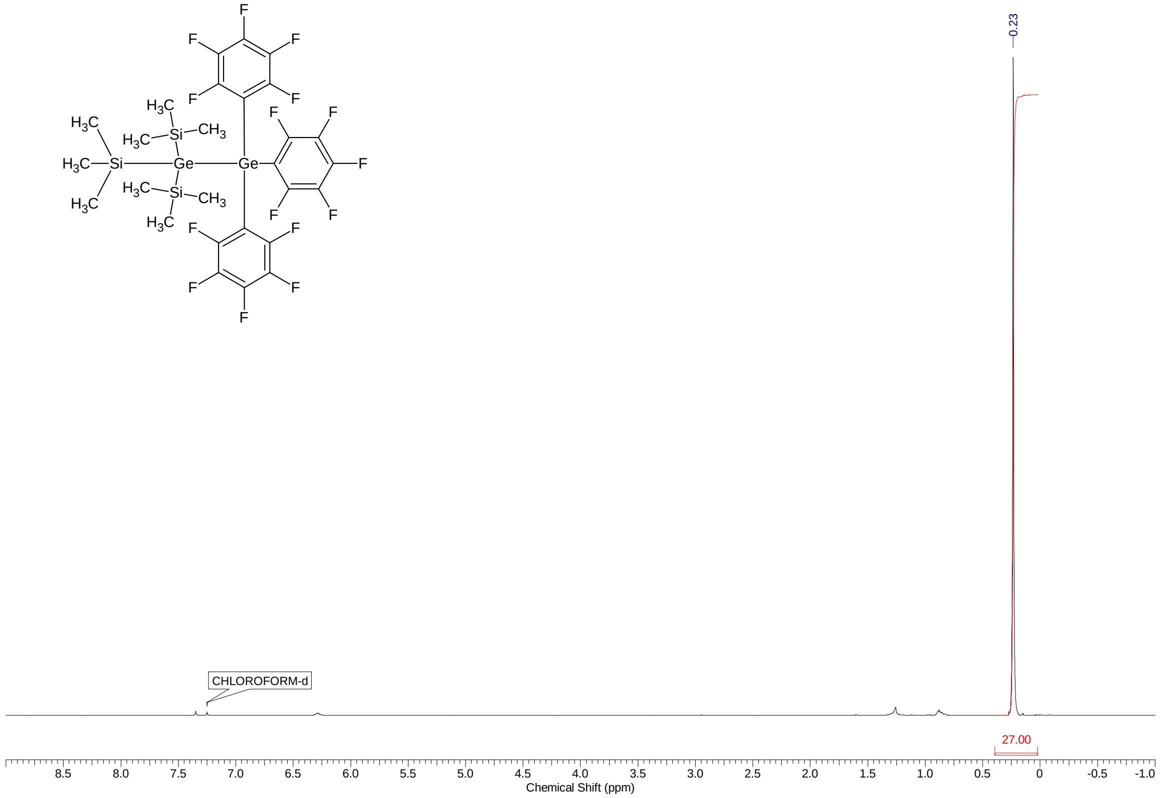

Fig. S8. ^{1}H NMR spectrum of $(Me_3Si)_3Ge\text{-}Ge(C_6F_5)_3$ (**3**) ($CDCl_3$, RT).

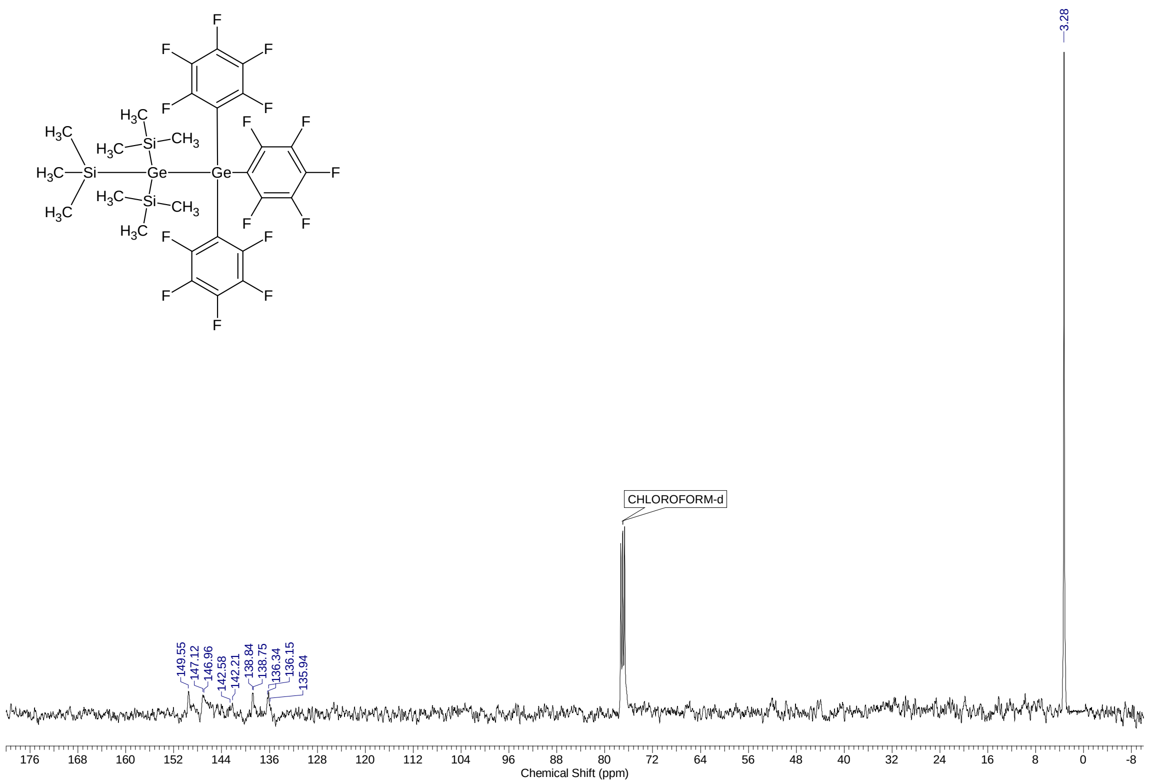

Fig. S9. ^{13}C NMR spectrum of $(Me_3Si)_3Ge$-$Ge(C_6F_5)_3$ (**3**) ($CDCl_3$, RT).

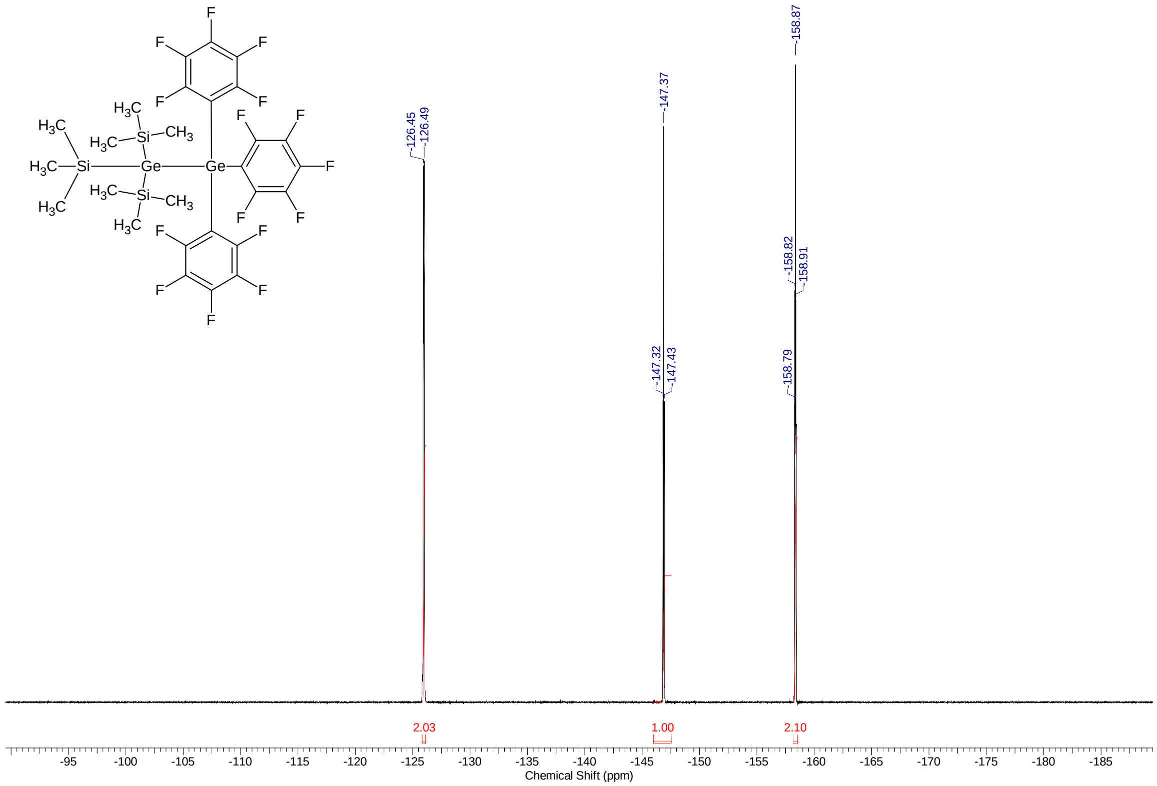

Fig. S10. ^{19}F NMR spectrum of $(Me_3Si)_3Ge$-$Ge(C_6F_5)_3$ (**3**) ($CDCl_3$, RT).

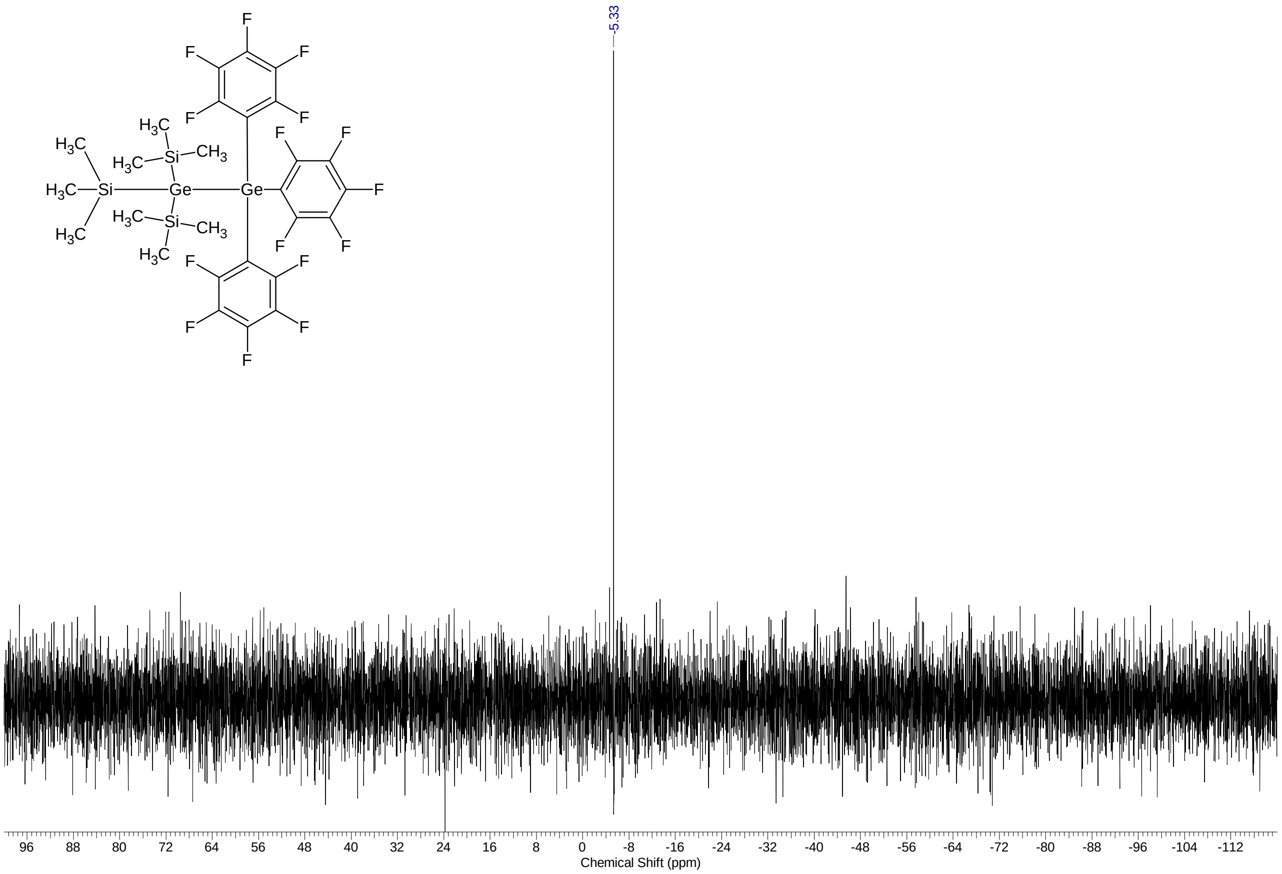

Fig. S11. ^{29}Si NMR spectrum of $(Me_3Si)_3Ge\text{-}Ge(C_6F_5)_3$ (**3**) ($CDCl_3$, RT).

UV/vis Absorption Spectra of the Compounds Obtained

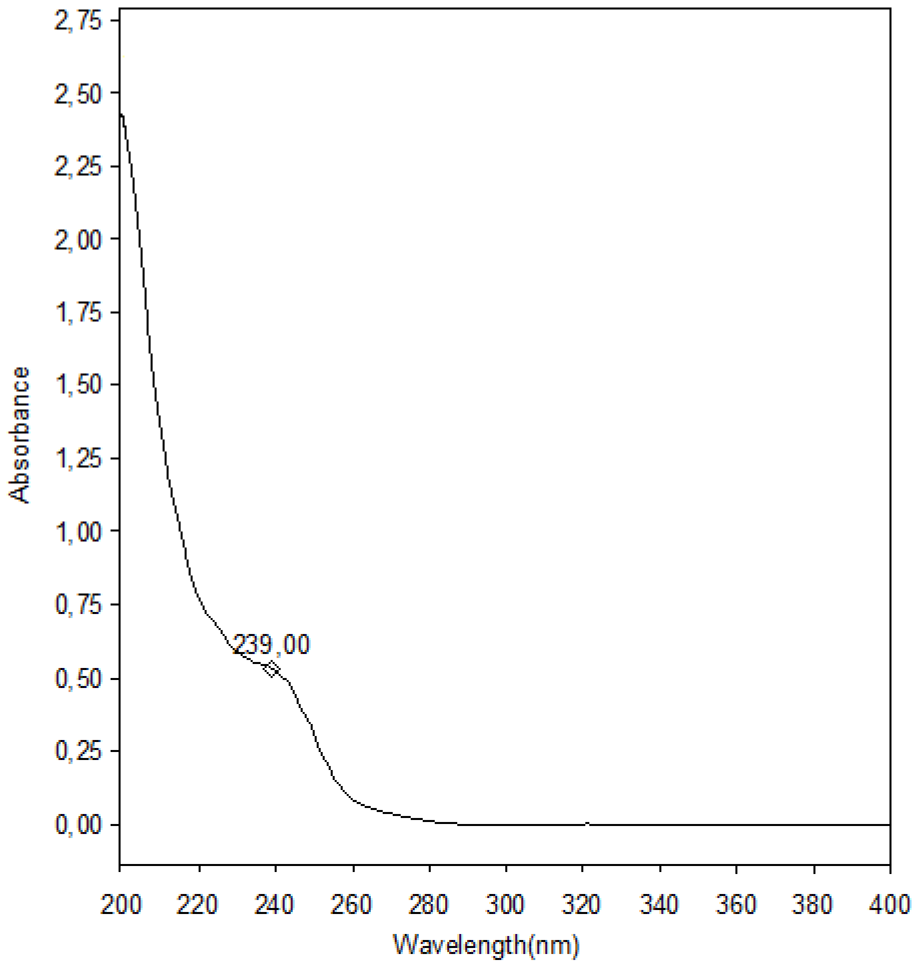

Batch : mar 4 C6H14 01 cm_03.sre

	A	B	C	D	E	F
1	mar 4 C6H14 01 c		1			
2	Cycle01	nm	239,00			
3	Manual	A	0,534			
4						
5						

Fig. S12. UV/vis Absorption Spectrum of $(Me_3Si)_3Ge$-$Ge(C_6H_4Me$-$p)_3$ (**1**) (*n*-hexane, RT).

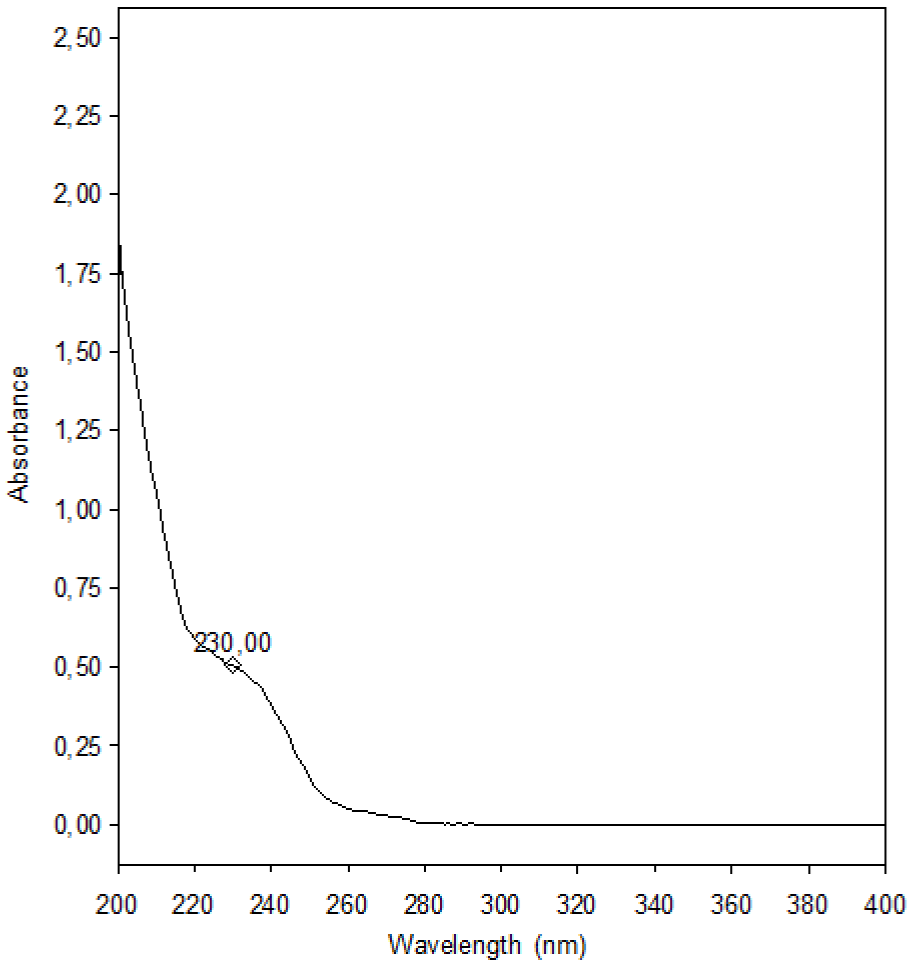

Batch : mar 5 C6H14 01 cm.sre

	A	B	C	D	E	F
1	**mar 5 C6H14 01 c**		**1**			
2	Cycle01	nm	230,00			
3	Manual	A	0,505			
4						
5						

Fig. S13. UV/vis Absorption Spectrum of $(Me_3Si)_3Ge\text{-}Ge(p\text{-}C_6H_4F)_3$ (**2**) (*n*-hexane, RT).

Data of the Single Crystal X-ray Diffraction Analysis

Table S1. Crystallographic data for complex $(Me_3Si)_3Ge\text{-}Ge(C_6H_4Me\text{-}p)_3$ (**1**) and $(Me_3Si)_3Ge\text{-}Ge(C_6H_4F\text{-}p)_3$ (**2**).

	$(Me_3Si)_3Ge\text{-}Ge(C_6H_4Me\text{-}p)_3$ (**1**)	$(Me_3Si)_3Ge\text{-}Ge(p\text{-}C_6H_4F)_3$ (**2**)
formula	$C_{30}H_{48}Ge_2Si_3$	$C_{27}H_{39}F_3Ge_2Si_3$
M_w	638.13	650.03
temperature (K)	150(2)	120(2)
size (mm)	0.25 x 0.20 x 0.06	0.24 x 0.20 x 0.18
crystal system	triclinic	triclinic
space group	*P-1*	*P-1*
a (Å)	10.3142(5)	12.6869(9)
b (Å)	12.5268(6)	16.0638(11)
c (Å)	14.3782(7)	16.0959(11)
α (deg)	92.799(1)	86.839(1)
β (deg)	99.352(1)	86.299(1)
γ (deg)	110.923(1)	81.912(1)
V (Å^3)	1700.61(14)	3237.4(4)
Z	2	4
P_{calcd}(g*cm^{-3})	1.246	1.334
abs. coeff. μ (MoKα) (mm^{-1})	1.889	1.999
F(000)	668	1336
θ range (deg)	2.22 – 28.00	1.76 – 29.00
no. of collected/unique rflns.	17699 / 8197	36739 / 17152
R_{int}	0.0147	0.0202
data/restraints/parameters	8197 / 0 / 329	17152 / 0 / 649
goodness of fit on F^2	1.030	1.052
final R indices (I> 2σ(I))	R_1 = 0.0219, wR_2 = 0.0583	R_1 = 0.0273, wR_2 = 0.0630
R indices (all data)	R_1 = 0.0264, wR_2 = 0.0603	R_1 = 0.0415, wR_2 = 0.0671
largest diff. peak/hole (e/Å^3)	-0.203 / 0.440	-0.285 / 0.377

Data of DFT calculations

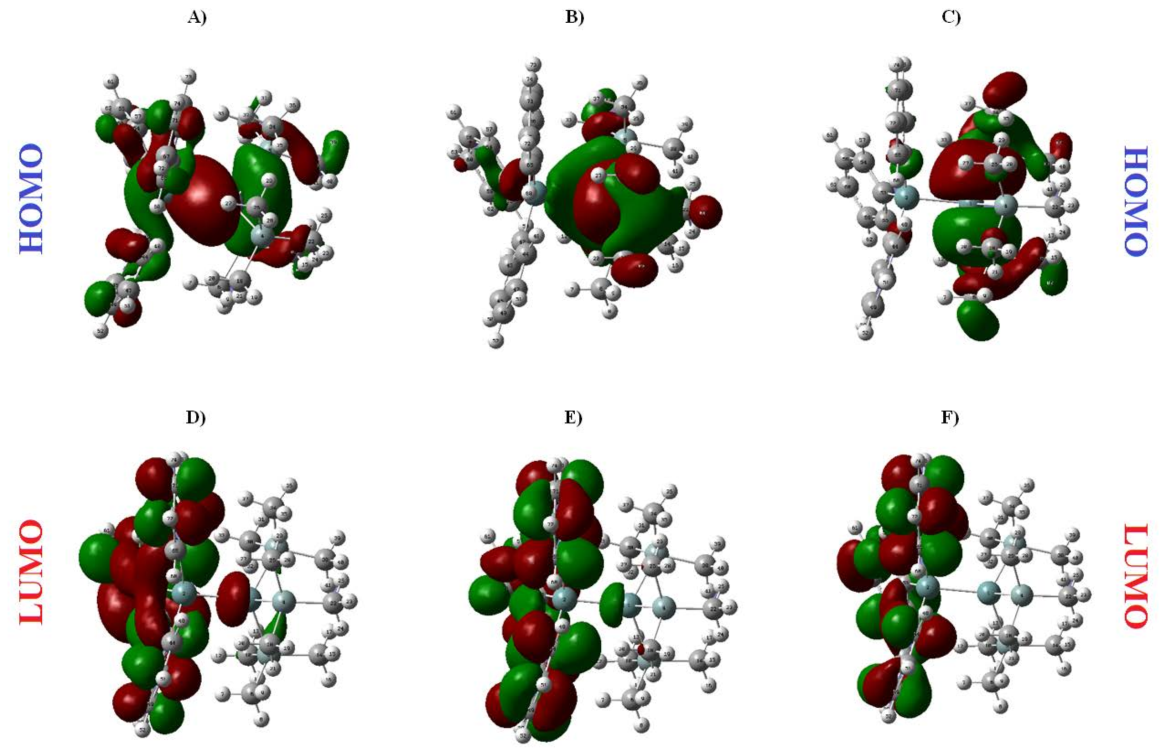

Fig. S14. Graphical representation of HOMO (**A**), HOMO-1 (**B**), HOMO-2 (**C**), LUMO (**D**), LUMO+1 (**E**), LUMO+2 (**F**) for compound $(Me_3Si)_3Ge\text{-}GePh_3$.
